# M-DNA/Transition Metal Dichalcogenide Hybrid Structure-based Bio-FET sensor with Ultra-high Sensitivity

**DOI:** 10.1038/srep35733

**Published:** 2016-10-24

**Authors:** Hyung-Youl Park, Sreekantha Reddy Dugasani, Dong-Ho Kang, Gwangwe Yoo, Jinok Kim, Bramaramba Gnapareddy, Jaeho Jeon, Minwoo Kim, Young Jae Song, Sungjoo Lee, Jonggon Heo, Young Jin Jeon, Sung Ha Park, Jin-Hong Park

**Affiliations:** 1 School of Electronic and Electrical Engineering, Sungkyunkwan University, Suwon 440-746, Korea; 2 Department of Physics, Sungkyunkwan University, Suwon 440-746, Korea; 3 SKKU Advanced Institute of Nanotechnology (SAINT), Sungkyunkwan University, Suwon 440-746, Korea; 4 Korea Advanced Nano Fab Center, Suwon 443-270, Korea

## Abstract

Here, we report a high performance biosensor based on (*i*) a Cu^2+^-DNA/MoS_2_ hybrid structure and (*ii*) a field effect transistor, which we refer to as a bio-FET, presenting a high sensitivity of 1.7 × 10^3^ A/A. This high sensitivity was achieved by using a DNA nanostructure with copper ions (Cu^2+^) that induced a positive polarity in the DNA (receptor). This strategy improved the detecting ability for doxorubicin-like molecules (target) that have a negative polarity. Very short distance between the biomolecules and the sensor surface was obtained without using a dielectric layer, contributing to the high sensitivity. We first investigated the effect of doxorubicin on DNA/MoS_2_ and Cu^2+^-DNA/MoS_2_ nanostructures using Raman spectroscopy and Kelvin force probe microscopy. Then, we analyzed the sensing mechanism and performance in DNA/MoS_2_- and Cu^2+^-DNA/MoS_2_-based bio-FETs by electrical measurements (I_D_-V_G_ at various V_D_) for various concentrations of doxorubicin. Finally, successful operation of the Cu^2+^-DNA/MoS_2_ bio-FET was demonstrated for six cycles (each cycle consisted of four steps: 2 preparation steps, a sensing step, and an erasing step) with different doxorubicin concentrations. The bio-FET showed excellent reusability, which has not been achieved previously in 2D biosensors.

Recently, biosensors have been applied in various fields such as the food industry^1^, medical diagnostics and treatment^2^, national security applicaitons^3^, and environmental monitoring^4^. Biosensors based on a field effect transistors (FETs) are of particular interest owing to their superior sensitivity, low power consumption, low fabrication cost, and excellent portability. In conventional FET-based biosensors (bio-FETs), the surface of the gate dielectric layer is functionalized by receptors for selectively capturing target molecules. This causes an electrostatic gating effect when the target molecules are captured. Therefore, the performance (sensitivity) of bio-FETs is strongly dependent on (*i*) the thickness and the dielectric constant (κ) of the gate dielectric and (*ii*) the quality of the interface between the channel and the gate dielectric. Three-dimensional (3D) semiconductors, such as silicon (Si) and Germanium (Ge)^5^, are typically used as channel materials because they have been used for conventional logic devices. However, these materials prevent the fabrication of highly sensitive bio-FET devices because 3D materials have relatively worse electrostatics compared to one- and two-dimensional (1D and 2D) materials. Since the 1D and 2D materials have inherently small body dimensions compared to 3D materials, when they are applied for transistor-like devices, the carriers in the 1D and 2D materials are expected to be better modulated by electrostatic effects, such as gate e-field in the transistor and positively/negatively charged target molecules in the biosensor. Although several bio-FETs with very high sensitivity^6^,^7^ were demonstrated on 1D materials, such as carbon nanotubes (CNTs) and Si nanowires (NWs), the feasibility of bio-FETs on 1D structures is still questionable because it is difficult to fabricate large-scale 1D devices in a cost effective manner.

From this point of view, transition metal dichalcogenide (TMD), an atomically thin 2D layer with a very low density of surface defect states (only point defects exist), is a very attractive material for future highly sensitive bio-FETs. TMD materials consist of 2D stacked layers, where adjacent layers with a very low surface defect density are weakly bound by van der Waals force and each layer is formed by covalently bonded transition metals and dichalcogenide atoms in a hexagonal lattice. Sarkar *et al*.^8^ were the first to report a 2D TMD-based bio-FET device with a HfO_2_ gate dielectric functionalized by APTES (3-aminoproplytriethoysilane) and biotin; this device showed very high sensitivity values of 713 for detection of pH and 196 for streptadavin. Recently, Lee *et al*.^9^ demonstrated another highly sensitive 2D bio-FET without a chemically treated gate dielectric layer, where a sensitivity of about 84 was obtained when detecting prostate specific antigen. A sensor based on a cyclodextrin-graphene hybrid nanosheet for the detection of doxorubicin (an anticancer medicine) was proposed by Guo *et al*.^10^ and they reported a ~1.4 sensitivity between 10 nM and 200 nM.

Here, we demonstrate a highly sensitive biosensor based on a Cu^2+^-DNA/MoS_2_ hybrid structure that employs the same operating principle as a FET. Here, “Cu^2+^-DNA” indicates a DNA nanostructure with Cu^2+^ ions. High sensitivity is achieved in the Cu^2+^-DNA/MoS_2_-based bio-FET because (*i*) there are many more sites in the backbone and base of Cu^2+^-DNA nanostructures for capturing target molecules (doxorubicin) than that of DNA alone and (*ii*) no high-κ insulating layer is used between the channel region and the receptor. We study the influence of doxorubicin on DNA/MoS_2_ and Cu^2+^-DNA/MoS_2_ structures (receptors) in detail with Raman spectroscopy, Kelvin probe force microscopy (KPFM), and electrical measurement (I_D_-V_G_). The sensing mechanism and performance are then discussed for both DNA/MoS_2_- and Cu^2+^-DNA/MoS_2_-based bio-FETs by varying the concentrations of doxorubicin (target). Finally, we confirm the reusability of the Cu^2+^-DNA/MoS_2_-based bio-FETs by repeating preparation, sensing, and erasing steps, which has not yet been achieved in previous 2D bio-FETs.

## Result and Discussions

### Synthesis and analysis of DNA nanostructures with incorporated Cu^2+^ ions and doxorubicin

**Figure 1:**
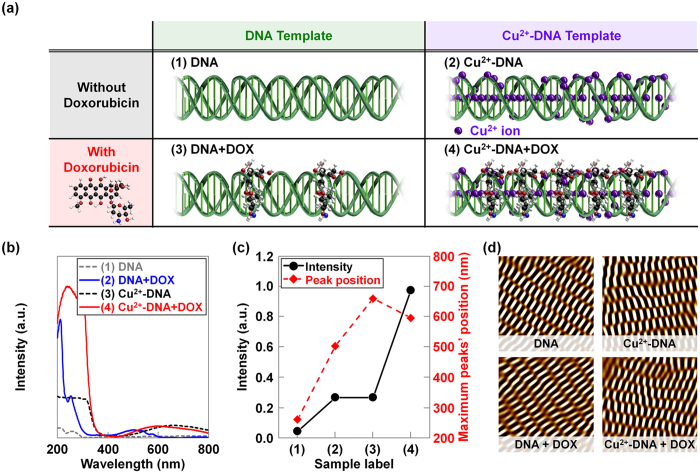
Synthesis and analysis of DNA nanostructures with incorporated Cu^2+^ ions and doxorubicin. (**a**) The schematics of (1) DX DNA, (2) Cu^2+^ ions coordinated in DNA, (3) doxorubicin coordination in DNA, and (4) the combination of Cu^2+^ ions and doxorubicin coordination in DNA nanostructures. (**b**) The absorbance spectra and (**c**) the analysis (intensity and maximum peak positions) of the absorption peaks, which were obtained on various DNA nanostructures with doxorubicin, Cu^2+^ ions, and the combination of Cu^2+^ ions and doxorubicin. For reference, the absorption peak of pristine DNA is located at about 260 nm. (**d**) The noise-filtered 2D spectrum AFM images constructed by using a fast Fourier transform on DNA, DOX-DNA, Cu^2+^-DNA, and Cu^2+^-DOX-DNA lattices, respectively.

DNA double-crossover (DX) nanostructures were fabricated by the conventional free solution annealing method. A unit DX tile was organized in two DX junctions, and two parallel duplexes were tied up. Two types of DX tiles were then used to construct DX DNA nanostructures^11^,^12^,^13^,^14^. Figure 1a shows the schematic diagrams of (1) the DNA nanostructure, (2) Cu^2+^ ion coordination in the DNA, (3) doxorubicin coordination in DNA, and (4) the combination of Cu^2+^ ions and doxorubicin coordination in DNA nanostructures. In previous reports, we studied the concentration dependent physical characteristics of various ions and doxorubicin coordinated DNA molecules in order to estimate the optimum concentration for structural stability and determine significant changes in functionalities^13^,^14^,^15^,^16^,^17^,^18^,^19^. Considering the parameters required to maintain deformation-free DNA nanostructures, we added the appropriate amounts of Cu^2+^ ions and doxorubicin molecules into the DNA nanostructures. As shown in Fig. 1a, the Cu^2+^ ions became intercalated between base-pairs and bound onto the phosphate backbone sites because they were size compatible. The DNA nanostructures with Cu^2+^ ions were mostly stable up to 4 mM, but above this concentration the structures were not well formed. This can be explained according to specific and non-specific binding. While specific binding dominates at a low Cu^2+^ ion concentration in the DNA, non-specific binding also plays a role at a higher concentration. In the case of specific binding, the lattices are minimally deformed because Cu^2+^ ions are systematically coordinated with particular sites. In non-specific binding, the lattices may be more severely damaged and deformed because the Cu^2+^ ions are randomly coordinated within the DNA duplex. When we coordinated the doxorubicin into DNA, the binding of doxorubicin had periodic orientation *via* an intercalation mode between the nucleosides. Because doxorubicin has a rough plane shape and is larger than the single nucleotide, doxorubicin molecules are expected to reside between layers of base-pairs of DNA duplexes through chemical bonds with nucleosides, as shown in Fig. 1.

Figure 1b,c show the absorption spectra and the analysis (intensity and maximum peak positions) of the absorption peaks, which were obtained on various DNA nanostructures with doxorubicin, Cu^2+^ ions, and a combination of Cu^2+^ ions and doxorubicin. For reference, the absorption peak of pristine DNA is located at about 260 nm. The DNA absorption peak intensity increased with coordinating doxorubicin, Cu^2+^ ions, and a combination of Cu^2+^ ions and doxorubicin into DNA nanostructures. Additionally, new absorption peaks appeared at approximately 505 nm for doxorubicin, 660 nm for Cu^2+^ ions, and 596 nm for the combination of Cu^2+^ ions and doxorubicin into DX DNA nanostructures. Tremendous changes in intensity of the DNA absorption peaks and a significant shift in the new absorption peak position by the coordinating molecules indicate that the Cu^2+^ ions and doxorubicin were properly bound to the DNA nanostructures. The absorption peak intensity values of Cu^2+^ ions or doxorubicin coordinated DNA were much lower than that of DNA nanostructures with the combination of Cu^2+^ ions and doxorubicin. This interesting behavior indicates that the Cu^2+^ ions in DNA worked as mediators for binding more doxorubicin molecules into DNA nanostructures, because the opposite polarity induced strong interactions between positively charged Cu^2+^ ions and negatively charged doxorubicin molecules. Furthermore, we performed atomic force microscopy (AFM) to confirm the DX nanostructure formation with Cu^2+^ ions, doxorubicin, and a combination of Cu^2+^ ions and doxorubicin. In Fig. 1d, the noise-filtered 2D fast Fourier transform spectrum images show the periodicity of the unit building block (DX tile). The AFM images revealed that the surface morphology of pristine DNA and coordinated DNA nanostructures were similar, indicating that the coordinating molecules (Cu^2+^ ions and doxorubicin) did not damage the DNA nanostructures.

### The influence of receptor (DNA and Cu^2+^-DNA) and target (doxorubicin) molecules on MoS_2_

**Figure 2:**
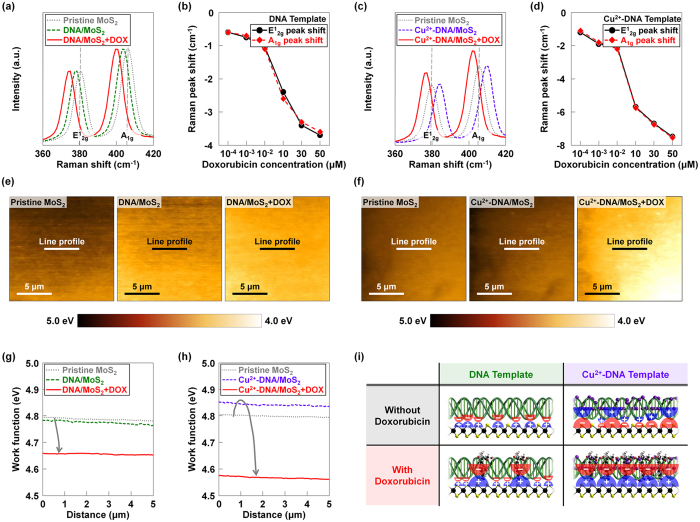
The influence of receptor (DNA and Cu^2+^-DNA) and target (doxorubicin) molecules on MoS_2_. Raman spectra taken on (**a**) pristine MoS_2_, DNA/MoS_2_ and (**c**) pristine MoS_2_, Cu^2+^-DNA/MoS_2_ before and after being exposed to 50 μM doxorubicin molecules. Extracted E^1^_2g_ and A_1g_ peaks of the MoS_2_ flakes coated by (**b**) DNA and (**d**) Cu^2+^-DNA nanostructures after being exposed to doxorubicin molecules with various concentrations (10^−4^ μM, 10^−3^ μM, 10^−2^ μM, 10 μM, 30 μM, and 50 μM). KPFM mapping images and work function values were respectively obtained on the (**e**) pristine MoS_2_ surface, DNA/MoS_2_ surface before and after being exposed to 50 μM doxorubicin molecules, and (**f**) pristine MoS_2_ surface, Cu^2+^-DNA/MoS_2_ surface before and after being exposed to 50 μM doxorubicin molecules. The work function values on the (**g**) pristine MoS_2_, DNA/MoS_2_ surface and (**h**) pristine MoS_2_, Cu^2+^-DNA/MoS_2_ before and after being exposed to 50 μM doxorubicin molecules. (**i**) The schematic diagrams presenting the predicted dipoles on DNA/MoS_2_ and Cu^2+^-DNA/MoS_2_ hybrid structures before and after doxorubicin molecules are detected.

To investigate the influence of (*i*) DNA/Cu^2+^-DNA nanostructures (receptors) and (*ii*) doxorubicin molecules (targets), Raman analysis was performed on the MoS_2_ flakes coated by DNA or Cu^2+^-DNA nanostructures before and after being exposed to doxorubicin molecules with various concentrations (10^−4^ μM, 10^−3^ μM, 10^−2^ μM, 10 μM, 30 μM, and 50 μM). Figure 2a shows the Raman spectra measured on pristine MoS_2_, DNA/MoS_2_, and DNA/MoS_2_ after being exposed to a 50 μM solution of doxorubicin molecules. The conventional peaks (E^1^_2g_ and A_1g_) were observed at 382 cm^−1^ and 408 cm^−1^ on pristine MoS_2_, which respectively indicate the in-plane and out-of-plane vibrations for bulk MoS_2_. After coating the pristine MoS_2_ with DNA nanostructures, the E^1^_2g_ and A_1g_ peaks were shifted towards the negative direction (ΔE^1^_2g_ = −1.9 cm^−1^ and ΔA_1g_ = −2.0 cm^−1^)^20^. This is because the DNA nanostructures have negative charges originating from the phosphate backbones (PO_4_^−^), and those subsequently attract holes in MoS_2_ to the interface region between DNA nanostructures and MoS_2_^21^,^22^. As shown in Fig. 2b, both peak shift values (ΔE^1^_2g_ and ΔA_1g_), which indicate differences between MoS_2_ Raman peak positions before and after detecting doxorubicin, were further shifted to the negative direction as the concentration of doxorubicin increased from 10^−4^ μM to 50 μM. We attributed this behavior to the negative charges from doxorubicin (originating from –OH functional groups), which become stronger on the MoS_2_ surface. The Raman spectra of pristine MoS_2_, Cu^2+^-DNA/MoS_2_, and Cu^2+^-DNA/MoS_2_ after being exposed to 50 μM doxorubicin are shown in Fig. 2c. The Cu^2+^-DNA nanostructures attract electrons in MoS_2_ and then hold them at the interface between Cu^2+^-DNA and MoS_2_, resulting in positive shifts of Raman peaks (ΔE^1^_2g_ = 5.1 cm^−1^ and ΔA_1g_ = 5.2 cm^−1^). When doxorubicin molecules were detected by the Cu^2+^-DNA, the shifted Raman peaks moved back in the negative direction. The degree of the positive shift was determined by the concentration of doxorubicin because of the –OH functional groups that had negative charges in the doxorubicin molecules. In the case of a low doxorubicin concentration between 10^−4^ μM and 10^−2^ μM, the peak shift values were relatively small (−1.1 to −2.1 cm^−1^) due to the existing positive charges from the Cu^2+^ ions, as seen in Fig. 2d. However, above 10 μM of doxorubicin, the peaks were more negatively shifted as the Cu^2+^ ions were overwhelmed by doxorubicin, resulting in high negative peak shift values (ΔE^1^_2g_ = −7.5 cm^−1^ and ΔA_1g_ = −7.5 cm^−1^). In the Cu^2+^-DNA/MoS_2_ hybrid structure, we observed relatively larger shifts in both E^1^_2g_ and A_1g_ peaks about −1.1 and −1.2 cm^−1^ (10^−4^ μM), −1.8 and −1.9 cm^−1^ (10^−3^ μM), −2.2 and −2.1 cm^−1^ (10^−2^ μM), −5.8 and −5.7 cm^−1^ (10 μM), −6.8 and −6.7 cm^−1^ (30 μM), and −7.6 and −7.5 cm^−1^ (50 μM), compared to the case of DNA/MoS_2_ hybrid structure. In order to confirm the change of work function in MoS_2_ flakes coated by DNA or Cu^2+^-DNA nanostructure before and after being exposed to doxorubicin molecules, we also conducted Kelvin probe force microscopy analysis. Before the measurement, KPFM tip was calibrated on a highly oriented pyrolytic graphite (HOPG) surface. Then, we obtained the work function (*W*) values from the contact potential difference (Δ*V*_CPD_) between the KPFM tip and MoS_2_ surface. Figure 2e shows the work function mapping images taken on the surfaces of the MoS_2_ flakes coated by DNA nanostructure before and after being exposed to doxorubicin molecules. After coating the pristine MoS_2_ with DNA nanostructures, the brighter image was observed, indicating that the work function of MoS_2_ was decreased. After being exposed to a 50 μM doxorubicin molecules, the image was brighter than that of DNA/MoS_2_ surface because the work function of MoS_2_ was further decreased by the negative charges from doxorubicin molecules. However, in the other case using Cu^2+^-DNA nanostructure (Fig. 2f), the darker image was obtained when compared to the pristine MoS_2_ surface after being coated by Cu^2+^-DNA nanostructures, because the work function was increased by the positive charges in Cu^2+^-DNA. After doxorubicin molecules were detected by the Cu^2+^-DNA, the image became brighter, even compared to the case that doxorubicin molecules were detected by DNA nanostructures. Figure 2g shows the work function values extracted on the pristine MoS_2_, DNA/MoS_2_, and DNA/MoS_2_ after being exposed to a 50 μM solution with doxorubicin molecules. The work function values were extracted at the specific line in MoS_2_ KPFM images, where the decreasing trend was observed (*W*_pristine MoS2_ = 4.79 eV and *W*_DNA/MoS2_ = 4.77 eV) due to the negative charges originating from the phosphate backbones (PO_4_^−^). After being exposed to 50 μM doxorubicin solution, the work function values were further decreased (*W*_DNA/MoS__2+__DOX_ = 4.65 eV) because of the negative charges from doxorubicin molecules. In the case of MoS_2_ coated by Cu^2+^-DNA nanostructures, work function value was increased (*W*_pristine MoS2_ = 4.80 eV and *W*_Cu2+-DNA/MoS2_ = 4.85 eV) because the positive polarity of Cu^2+^ ions attracted the electrons. When doxorubicin molecules were detected by the Cu^2+^-DNA, the lower work function value was obtained (*W*_Cu2+-DNA/MoS__2+__DOX_ = 4.58 eV) due to the –OH functional groups with negative polarity in the doxorubicin molecules, which was even lower than the case of the DNA/MoS_2_ hybrid structure. Based on these Raman and KPFM analyses, the receptor template consisting of Cu^2+^-DNA is expected to further improve the ability to detect doxorubicin, compared to the DNA receptor template. Figure 2i presents schematic diagrams of DNA/MoS_2_ and Cu^2+^-DNA/MoS_2_ hybrid structures before and after doxorubicin molecules were detected, which also explain how the detected doxorubicin molecules affect the MoS_2_ layers *via* the DNA and Cu^2+^-DNA nanostructures. In the Cu^2+^-DNA/MoS_2_ hybrid structure, more doxorubicin molecules were attached onto the phosphate backbone (PO_4_^−^) sites of DNA *via* Cu^2+^ ions and intercalated in the base pairings, compared to the case of the DNA/MoS_2_ hybrid structure. Therefore, the Cu^2+^-DNA/MoS_2_ hybrid structure presented a higher sensitivity for doxorubicin.

### Characteristics of DNA/MoS_2_-and Cu^2+^-DNA/MoS_2_-based bio-FETs

**Figure 3:**
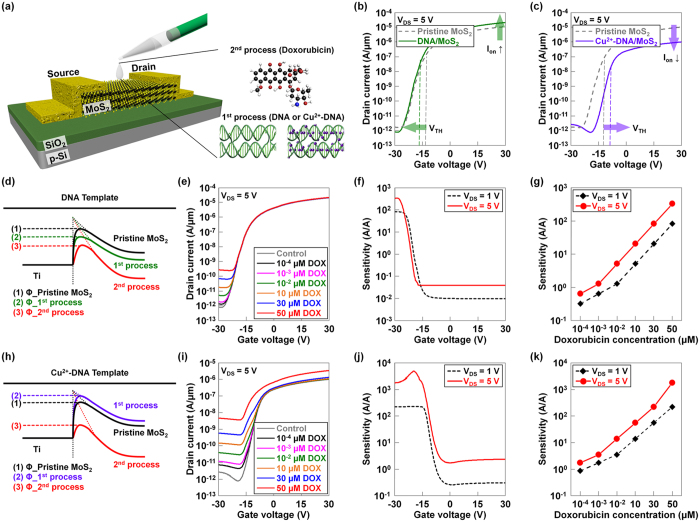
Characterization of DNA/MoS_2_- and Cu^2+^-DNA/MoS_2_-based bio-FETs. (**a**) Schematic diagrams of back-gate type FETs, and the process of detecting doxorubicin molecules. I_D_-V_G_ characteristics of MoS_2_ bio-FETs before and after coating the (**b**) DNA or (c) Cu^2+^-DNA nanostructures. The energy band diagrams at Ti/MoS_2_ junctions of (**d**) pristine MoS_2_, DNA/MoS_2_ (1^st^ process), and DNA/MoS_2_ after detecting doxorubicin (2^nd^ process) and (**h**) pristine MoS_2_, Cu^2+^-DNA/MoS_2_ (1^st^ process), and Cu^2+^-DNA/MoS_2_ after detecting doxorubicin (2^nd^ process). I_D_-V_G_ characteristics of (**e**) DNA/MoS_2_- and (i) Cu^2+^-DNA/MoS_2_-based bio-FETs according to various concentrations of doxorubicin (10^−4^ μM, 10^−3^ μM, 10^−2^ μM, 10 μM, 30 μM, and 50 μM). Calculated sensitivity ((I_off_after sensing_ − I_off_before sensing_)/I_off_before sensing_) of (**f**) DNA/MoS_2_- and (**j**) Cu^2+^-DNA/MoS_2_-based bio-FETs as a function of gate voltage at different drain voltages when detecting 50 mM of doxorubicin. The sensitivity of (**g**) DNA/MoS_2_- and (**k**) Cu^2+^-DNA/MoS_2_-based bio-FETs as a function of the concentration of doxorubicin at different drain voltages.

Next, we investigated the performance of DNA/MoS_2_- and Cu^2+^-DNA/MoS_2_-based bio-FETs as a function of the concentration of doxorubicin and the applied drain voltage. As seen in Fig. 3a, back-gate type FETs were fabricated on MoS_2_ layers, followed by coating process of DNA or Cu^2+^-DNA nanostructures onto the channel regions, eventually achieving DNA/MoS_2_ or Cu^2+^-DNA/MoS_2_ hybrid structures for doxorubicin detection. To electrically confirm the effects of the DNA or Cu^2+^-DNA nanostructures on MoS_2_ layers, we performed current-voltage (I_D_-V_G_) measurements. Figure 3b,c show I_D_-V_G_ characteristics of MoS_2_ bio-FETs before and after coating the DNA or Cu^2+^-DNA nanostructures. After coating the DNA nanostructures on MoS_2_, the on-current (at V_GS_ = 30 V) slightly increased from 1.1 × 10^−5^ A/μm (pristine MoS_2_) to 2.1 × 10^−5^ A/μm (DNA/MoS_2_), and the threshold voltage was negatively shifted from −13.2 V (pristine MoS_2_) to −15.5 V (DNA/MoS_2_). This is because the hole carriers accumulated at the interface between DNA and MoS_2_ owing to the negative charges in the phosphate backbone (PO_4_^−^) of the DNA nanostructures, resulting in an n-doping phenomenon in MoS_2_. In contrast, after the Cu^2+^-DNA nanostructure was coated on MoS_2_, the on-current (at V_GS_ = 30 V) decreased from 1.1 × 10^−5^ A/μm (pristine MoS_2_) to 1.0 × 10^−6^ A/μm (Cu^2+^-DNA/MoS_2_), and the threshold voltage positively shifted from −12.8 V (pristine MoS_2_) to −9.8 V (Cu^2+^-DNA/MoS_2_). Unlike the case of DNA nanostructures on MoS_2_, electrons accumulated and were held at the interface between Cu^2+^-DNA and MoS_2_ because of the positive polarity of Cu^2+^ ions in the Cu^2+^-DNA nanostructure (p-doping of MoS_2_). Figure 3d shows the energy band diagrams at the Ti/MoS_2_ junctions for the three cases of pristine MoS_2_, DNA/MoS_2_ (1^st^ process), and DNA/MoS_2_ after detecting doxorubicin (2^nd^ process). After the DNA nanostructures were coated on pristine MoS_2_, the Ti/MoS_2_ junction had a lower effective electron barrier height for electron injection. This effective barrier height decreased further when doxorubicin was detected by the DNA/MoS_2_ hybrid structure because of the negative charges originating from DNA nanostructures and doxorubicin. Figure 3e shows the I_D_-V_G_ characteristics of DNA/MoS_2_-based bio-FET for various concentrations of doxorubicin. Since the effective electron barrier height was reduced as the concentration of doxorubicin increased from 10^−4^ μM to 50 mM, an increase in off-current (from 1.4 × 10^−12^ A/μm to 2.7 × 10^−10^ A/μm) was observed. Then, we calculated sensitivity, (I_off_after sensing_ − I_off_before sensing_)/I_off_before sensing_, as a function of gate voltage at different drain voltages when detecting 50 mM of doxorubicin, as shown in Fig. 3f. Here, a very high sensitivity of 82.5–325.9 A/A was observed at a V_GS_ of about −30 V because a much lower base current (8.4 × 10^−13^ A/μm) was obtained in the off-state as compared to other V_GS_ regions higher than −22 V. A higher V_DS_ resulted in a little bit higher sensitivity because a higher electric field occurred in the MoS_2_ region under a biased condition. This consequently increased the probability of collecting injected electrons when doxorubicin was introduced. Figure 3g presents the sensitivity of the DNA/MoS_2_-based bio-FET as a function of the concentration of doxorubicin at different drain voltages. The sensitivity at V_DS_ = 5 V increased from 0.6 to 325.9 A/A as the concentration of doxorubicin increased from 10^−4^ μM to 50 mM; these sensitivity values were higher than the sensitivity values at V_DS_ = 1V for all concentrations. Overall, in the DNA/MoS_2_ bio-FET, we obtained a high sensitivity of 325.9 A/A through a proper voltage bias condition (a more negative V_GS_ for a lower base current and a higher V_DS_ for better electron collection).

Similarly, Fig. 3h presents an energy band diagram of Ti/MoS_2_ junctions for another three samples: pristine MoS_2_, Cu^2+^-DNA/MoS_2_ (1^st^ process), and Cu^2+^-DNA/MoS_2_ after detecting doxorubicin (2^nd^ process). Unlike the case of the DNA nanostructure on MoS_2_, the effective electron barrier height was higher than that of pristine MoS_2_ when the Cu^2+^-DNA nanostructure was introduced onto the MoS_2_. Since the Cu^2+^ ions bonded with phosphate backbones and base pairings provide a positive polarity, electron carriers were held at the interface between Cu^2+^-DNA and MoS_2_. Because of this p-doping effect, the energy band was up-shifted compared to the pristine MoS_2_, eventually increasing the effective electron barrier height. When doxorubicin was detected by the Cu^2+^-DNA/MoS_2_ hybrid structure, negative charges from doxorubicin molecules seemed to compensate for the strength of the Cu^2+^ positive ions, subsequently shifting down the MoS_2_ energy band and increasing the electric field at the Ti/MoS_2_ junction. This phenomenon consequently reduced the effective barrier height of the Ti/MoS_2_ junction, eventually increasing the current (i.e., a Schottky barrier lowering effect). The I_D_-V_G_ characteristics of Cu^2+^-DNA/MoS_2_-based bio-FETs for various concentrations of doxorubicin are plotted in Fig. 3i, where the off-current at V_GS_ = −30 V increased significantly from 7.2 × 10^−12^ to 4.6 × 10^−9^ A/μm in the same doxorubicin concentration range (between 10^−4^ μM and 50 mM) as compared to the case of the DNA/MoS_2_-based bio-FET. This is because the Cu^2+^ ions incorporated into the DNA improved the ability of the DNA-based receptors to detect negatively polarized doxorubicin. In this case, phosphate backbone (PO_4_^−^) sites allowed capture of doxorubicin *via* the Cu^2+^ ions in addition to the base parings. As shown in Fig. 3j, the corresponding sensitivity was also calculated as a function of gate voltage at different drain voltages, where we obtained very high sensitivity in a much wider V_GS_ region (maximum 219.3 A/A at V_DS_ = 1 V and maximum 1757.1 A/A at V_DS_ = 5 V under V_GS_ = −14 V). The sensitivity was roughly five times higher than that of the DNA/MoS_2_-based bio-FET. This increased operating region (V_GS_ < −14V) of the bio-FET was attributed to an increase in electron barrier height by the Cu^2+^-DNA-based p-doping effect and the subsequent positive shift in V_TH_. In addition, like the case of the DNA/MoS_2_ hybrid structure, an 8-fold higher sensitivity was observed at higher V_DS_ because electron carriers injected after detecting doxorubicin were also expected to be more easily collected under stronger electric fields at higher V_DS_. Figure 3k shows the sensitivity of the Cu^2+^-DNA/MoS_2_ based bio-FET as a function of the doxorubicin concentration at different drain voltages. Here, we confirmed that the sensitivity increased from 1.7 to 1757.1 A/A at V_DS_ = 5 V as a function of doxorubicin concentration, which was also higher than the values when V_DS_ = 1 V was applied. Additionally, we investigated the long-term stability of the Cu^2+^-DNA/MoS_2_ based bio-FET by exposing the device to air for two weeks (Supporting information Figure S3).

### Reusability of Cu^2+^-DNA/MoS_2_ based-biosensor

**Figure 4:**
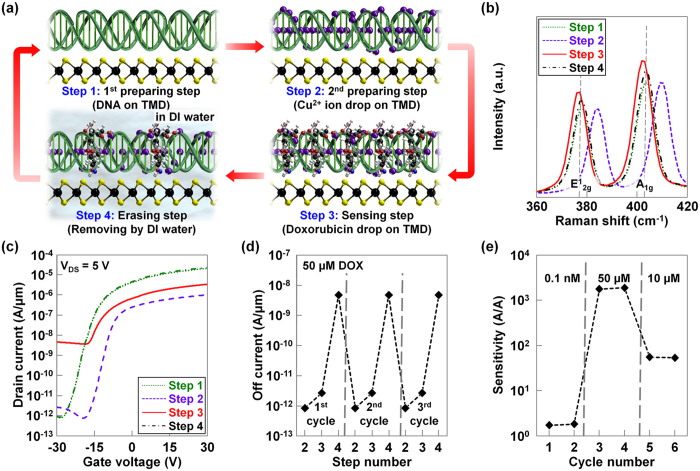
Reusability of Cu^2+^-DNA/MoS_2_ based-biosensor. (**a**) Schematic diagrams showing the detecting steps of Cu^2+^-DNA/MoS_2_-based bio-FET (1^st^/2^nd^ preparing, sensing, and erasing steps). (**b**) Raman spectra of the MoS_2_ measured in the four detecting steps. (**c**) I_D_-V_G_ characteristics of Cu^2+^-DNA/MoS_2_-based bio-FET in the four detecting steps. (**d**) Extracted off-current values at V_GS_ = −30 V in each step for three detecting cycles. (**e**) Sensitivity values of Cu^2+^-DNA/MoS_2_-based bio-FETs as a function of detecting cycles with different concentrations of doxorubicin.

Finally, we investigated the reusability of the Cu^2+^-DNA/MoS_2_ hybrid structure-based bio-FETs by repeating the preparation, sensing, and erasing steps. Figure 4a shows a schematic diagram that graphically explains the 1^st^/2^nd^ preparation, sensing, and erasing steps. In the first step, DNA nanostructures were coated and dried several times on a MoS_2_ layer to cover the surface of MoS_2_ (1^st^ preparation step), followed by the addition of Cu^2+^ ions (2^nd^ preparation step) for improving the doxorubicin detecting ability. Then, various concentrations of doxorubicin were dropped onto the bio-FET device and I_D_-V_G_ measurement was performed (3^rd^ sensing step). After sensing, the used doxorubicin molecules and Cu^2+^ ions were removed by deionized (DI) water to reuse the bio-FET with DNA receptor template (4^th^ erasing step). Because DI water rinsing does not destroy the DNA/MoS_2_ hybrid structure (Cu^2+^ ions and doxorubicin are only soluble in DI water), we started the second detection cycle by again transferring Cu^2+^ ions onto the DNA/TMD bio-FET (2^nd^ preparation step). Then, another sensing process was performed, and the used doxorubicin molecules and Cu^2+^ ions were removed again using the DI water rinsing process. In this way, the proposed Cu^2+^-DNA/MoS_2_ bio-FET can be used repeatedly, and it is also possible to detect various target molecules with one device if the molecules have negative polarity. Figure 4b presents Raman spectra of the MoS_2_ measured over four detecting steps. After the Cu^2+^ ions were dropped on the DNA/MoS_2_, both E^1^_2g_ and A_1g_ peaks in MoS_2_ shifted in the positive direction as already confirmed in the previous Raman analysis of Cu^2+^-DNA/MoS_2_. Then, the E^1^_2g_ and A_1g_ peaks were moved in the negative direction by the negative polarity of the doxorubicin, which compensated for the effect of the Cu^2+^ ions. After the fourth erasing step, the position of E^1^_2g_ and A_1g_ peaks returned to their original position (i.e., the position observed for DNA on MoS_2_). The I_D_-V_G_ characteristics of Cu^2+^-DNA based bio-FET were also investigated in the four detecting steps, as shown in Fig. 4c. We confirmed that the overall change in current level was similar to previous results and the current level returned to its initial shape after the 4^th^ erasing step. Then, we extracted the off-current values at V_GS_ = −30 V in each detecting cycle. These data are presented in Fig. 4d, where the change in off-current level in each step was repeated in all detecting cycles. For reference, this reusability analysis was repeated for ten detecting cycles and these results can be found in the Supporting information Figure S4. Finally, we changed the concentration of doxorubicin in six detecting cycles and then plotted the extracted sensitivity values in Fig. 4e. We first flowed a low concentration of doxorubicin (10^−4^ μM) in the first two cycles, and then increased the concentration up to 50 μM. As a result, a sensitivity of approximately 1.7, which was observed in the first two cycles, dramatically increased to 1.7 × 10^3^ in the 3^rd^ and 4^th^ cycles. In the final two cycles, the sensitivity was reduced down to about 54 because we lowered the concentration to 10 μM. Based on the observation that the sensitivity was almost same when the same concentration of doxorubicin was used, the proposed Cu^2+^-DNA/MoS_2_ bio-FET seems to provide reproducible results. We also compared our work with the previously reported biosensors in terms of materials, type of biosensor, detection molecules/range, sensitivity, and reusability (Supporting information Table S1).

## Conclusions

In conclusion, we demonstrated a Cu^2+^-DNA/MoS_2_ based bio-FET with an extremely high sensitivity of 1.7 × 10^3^ A/A. The high sensitivity was accomplished because the Cu^2+^-DNA nanostructures had a positive polarity, and these improved the detecting ability for doxorubicin-like molecules with negative polarity as compared to the DNA nanostructures alone. In addition, the short distance between the biomolecules and the sensor surface, which could be achieved without using high-κ dielectric layers in bio-FETs, also contributed to the high sensitivity. We first found the optimum conditions for the formation of Cu^2+^-DNA by considering the deformation phenomenon of Cu^2+^-DNA according to the concentration of doxorubicin. We then studied the influence of doxorubicin on Cu^2+^-DNA/MoS_2_ structure using Raman spectroscopy and Kelvin probe force microscopy. Based on these basic studies about the relationship between Cu^2+^-DNA/MoS_2_ hybrid structures and doxorubicin molecules, the operating principle of the Cu^2+^-DNA/MoS_2_-based bio-FET was then analyzed in detail under various concentrations of doxorubicin by performing the electrical measurements (I_D_-V_G_ at various V_D_). Finally, we confirmed the successful operation of the Cu^2+^-DNA/MoS_2_ bio-FET for six cycles with different doxorubicin concentrations (each cycle consists of four steps; 1^st^ and 2^nd^ preparation, 3^rd^ sensing, and 4^th^ erasing steps). This excellent reusability has not been reported previously for 2D biosensors.

## Experimental Methods

### Fabrication of DX DNA Lattice

High-performance liquid chromatography purified synthetic oligonucleotides of DNA strands were purchased from Bioneer (Daejeon, Korea). Two DX tiles were used for the fabrication of a DX DNA nanostructure using a conventional free solution annealing process. Complexes were formed by mixing a stoichiometric quantity of each strand in physiological 1 × TAE/Mg^2+^ buffer (40 mM Tris base, 20 mM acetic acid, 1 mM EDTA (pH 8.0), and 12.5 mM magnesium acetate) to form the DX structure. The complexes were cooled slowly from 95 to 25 °C to facilitate hybridization after placing the microtubes in 2 L of boiling water in a Styrofoam box for at least 24 h. The final concentration of DX DNA lattices was 200 nM.

### Absorbance Measurements

A Varian Cary 5G spectrophotometer was used to conduct the optical absorbance measurements of the DNA, DNA with doxorubicin, Cu^2+^-DNA, and Cu^2+^-DNA with doxorubicin in solution form (wavelengths between 800 and 200 nm). The spectrophotometer was equipped with two light sources: a deuterium arc lamp (near-infrared and visible) and a quartz W− halogen lamp (ultraviolet). It has also two detectors: a cooled PbS detector for the near-infrared region and a photomultiplier tube for the visible and ultraviolet regions. The spectrophotometer measure the frequency-dependent light intensity passing either through a vacuum or through the sample.

### AFM Measurements

AFM images were taken by pipetting 5 μL of the solutions on freshly cleaved mica, after which 30 μL of 1× TAE/Mg^2+^ buffer was pipetted onto the mica surface and another 10 μL of 1× TAE/Mg^2+^ buffer was dropped onto the AFM tip (Veeco Inc.). All AFM images were obtained on a Digital Instruments Nanoscope III (Veeco, USA) with a multimode fluid cell head in tapping mode using NP-S oxide-sharpened silicon nitride tips (Veeco, USA).

### Characterization of DNA/MoS_2_ or Cu^2+^-DNA/MoS_2_ without and with doxorubicin

First, DNA or Cu^2+^-DNA/MoS_2_/SiO_2_/Si samples were investigated and compared with a control sample (MoS_2_/SiO_2_/Si). Then, the DNA or Cu^2+^-DNA/MoS_2_/SiO_2_/Si samples with doxorubicin were measured using PL/Raman spectroscopy (Alpha300M +, WITec). Here, TMD bulk flakes with similar thicknesses (~32 nm) were selected to avoid the thickness effect. Raman spectroscopy with an excitation wavelength of 532 nm was used, where the laser beam size was approximately 0.7–0.9 μm, and the instrumental spectral resolution was less than 0.9 cm^−1^. An integration time of 5 seconds and a spectrometer with 1800 grooves/mm were used. For the KPFM measurement, a platinum/iridium (Pt/Ir) coated Si tip was used and the tip was calibrated on a HOPG surface. First, we calculated the work function of the KPFM tip (*W*_tip_ − *W*_HOPG_ = Δ*V*_CPD_HOPG_) using the well-known work function of the HOPG (*W*_HOPG_ = 4.6 eV) and the contact potential difference between tip and HOPG (Δ*V*_CPD_HOPG_ = 324 meV). We then found the work function of the TMD layers with the calculated *W*_tip_ (4.92 eV) value and the measured Δ*V*_CPD_TMD_ between KPFM tip and TMD surface (*W*_tip_ − *W*_TMD_ = Δ*V*_CPD_TMD_).

### Fabrication and electrical characterization of DNA/MoS_2_- and Cu^2+^-DNA/MoS_2_-based bio-FETs

For the fabrication of back-gated MoS_2_ transistors, source/drain electrode regions were patterned (channel length and width were 5 μm) on MoS_2_/SiO_2_/Si samples by optical lithography, followed by deposition of 10-nm-thick Ti and 50-nm-thick Au in an e-beam evaporator. Back-gated MoS_2_ transistors were coated by DNA or Cu^2+^-DNA and were electrically analyzed using an HP 4155A semiconductor parameter analyzer (I_D_-V_G_). The threshold voltage (V_TH_) and sensitivity, which is calculated as (I_off_after sensing_ − I_off_before sensing_)/I_off_before sensing_, were calculated from the I_D_-V_G_ data. All drain currents (I_DS_) were normalized by the channel width (W).

## Additional Information

**How to cite this article**: Park, H.-Y. *et al*. M-DNA/Transition Metal Dichalcogenide Hybrid Structure-based Bio-FET sensor with Ultra-high Sensitivity. *Sci. Rep.* **6**, 35733; doi: 10.1038/srep35733 (2016).

## Supplementary Material

Supplementary Information
