# Supplementary material for: M-DNA/Transition Metal Dichalcogenide Hybrid Structure-based Bio-FET sensor with Ultra-high Sensitivity
Source: Sci Rep. 2016 Oct 24;6:35733. doi: 10.1038/srep35733 (PMC5075907; doi:10.1038/srep35733)
Supplement: Supplementary Information [file srep35733-s1.doc]

**Supplementary Information**

**M-DNA/Transition Metal Dichalcogenide Hybrid Structure-based Bio-FET sensor with Ultra-high Sensitivity**

Hyung-Youl Park^1,+^, Sreekantha Reddy Dugasani^2,3,+^, Dong-Ho Kang^1^, Gwangwe Yoo^1^, Jinok Kim^1^, Bramaramba Gnapareddy^2^, Jaeho Jeon^3^, Minwoo Kim^3^, Young Jae Song^3^, Sungjoo Lee^3^, Jonggon Heo^4^, Young Jin Jeon^4^, Sung Ha Park^2,3,*^, and Jin-Hong Park^1,*^

^1^School of Electronic and Electrical Engineering, Sungkyunkwan University, Suwon 440-746, Korea
^2^Department of Physics, Sungkyunkwan University, Suwon 440-746, Korea
^3^ SKKU Advanced Institute of Nanotechnology (SAINT), Sungkyunkwan University, Suwon 440-746, Korea
^4^Korea Advanced Nano Fab Center, Suwon 443-270, Korea

^+^ Equally contributed as the first author.

^E)^ Corresponding Authors’ Email Addresses: [jhpark9@skku.edu](mailto:jhpark9@skku.edu) (J.-H. Park) and sunghapark@skku.edu (S. H. Park).

**I_D_-V_G_ characteristics of DNA/MoS_2_- and Cu^2+^-DNA/MoS_2_-based bio-FETs.**


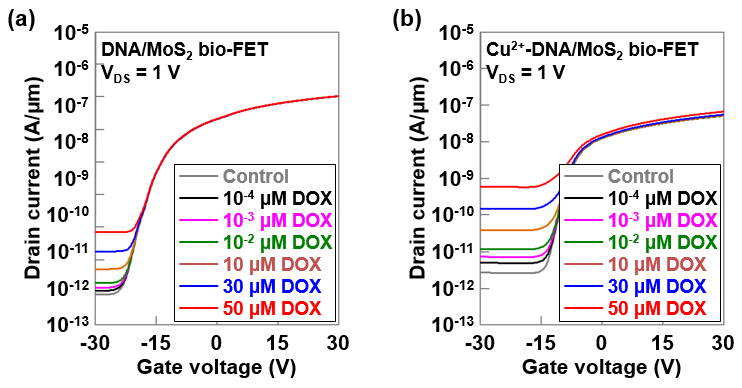


**Fig. S1.** I_D_-V_G_ characteristics (at V_DS_ = 1V) of (a) DNA/MoS_2_- (b) Cu^2+^-DNA/MoS_2_-based bio-FETs according to various concentrations of doxorubicin (10^-4^ μM, 10^-3^ μM, 10^-2^ μM, 10 μM, 30, μM, and 50 μM).

Fig. S1(a) shows the I_D_-V_G_ characteristics of DNA/MoS_2_-based bio-FET according to various concentrations of doxorubicin. The off-current increased from 8.4×10^-13^ to 6.9×10^-11^ A/μm as the concentration of doxorubicin increased from 10^-4^ μM to 50 mM because the effective electron barrier height was reduced at higher concentrations of doxorubicin. The I_D_-V_G_ characteristics of Cu^2+^-DNA/MoS_2_-based bio-FET with various concentrations of doxorubicin are plotted in Fig. S1(b). The off-current increased significantly (from 2.3×10^-12^ to 5.8×10^-10^ A/μm as the concentration of doxorubicin increased from 10^-4^ μM to 50 mM) as compared to the case of DNA/MoS_2_-based bio-FET. This is because the Cu^2+^ ions incorporated into the DNA improved the detecting ability of the DNA-based receptor for doxorubicin with negative polarity.

**I_D_-V_G_ characteristics of Cu^2+^-DNA/MoS_2_-based bio-FETs in each step for three detecting cycles**


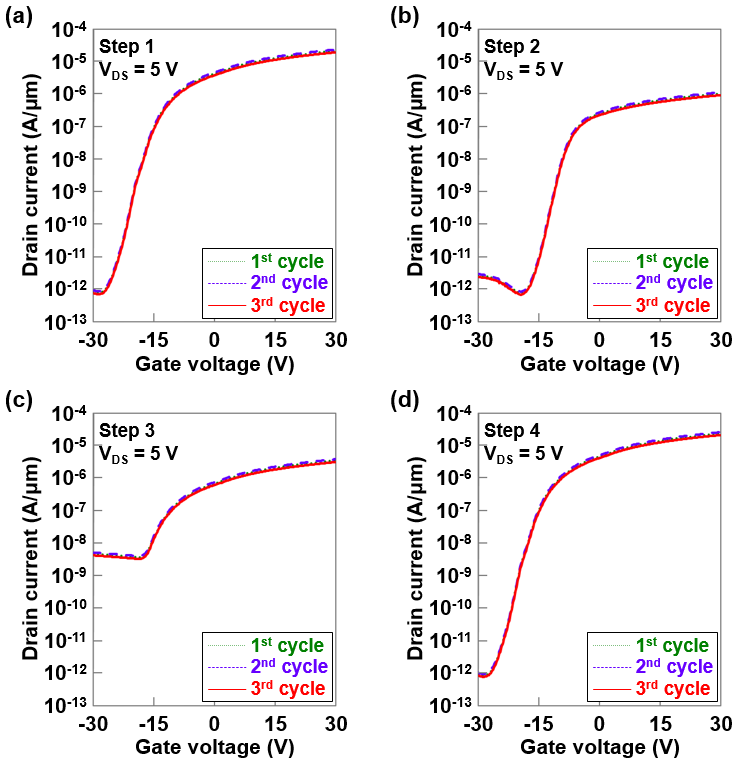


**Fig. S2.** I_D_-V_G_ characteristics of Cu^2+^-DNA/MoS_2_-based bio-FET in (a) 1^st^ preparation step, (b) 2^nd^ preparation step, (c) 3^rd^ sensing step, and (d) 4^th^ erasing step for three detecting cycles.

Fig. S2(a) presents the I_D_-V_G_ characteristics of the bio-FET after the DNA nanostructures were coated on the MoS_2_ for three detecting cycles (1^st^ preparation step). After the Cu^2+^ ions were dropped onto the DNA/MoS_2_-based bio-FET (2^nd^ preparation step), the on-current decreased from 2.1×10^-5^ A/μm (DNA/MoS_2_) to 1.0×10^-6^ A/μm (Cu^2+^-DNA/MoS_2_), and the threshold voltage was positively shifted from -15.5 V (DNA/MoS_2_) to -9.8 V (Cu^2+^-DNA/MoS_2_), as shown in Fig. S2(b). This is because the electrons were trapped at the interface between Cu^2+^-DNA and MoS_2_ due to the positive polarity of the Cu^2+^ ions in the Cu^2+^-DNA. As seen in Fig. S2(c), the off-current increased from 7.2×10^-12^ A/μm (before sensing) to 4.6×10^-9^ A/μm (after sensing) in 3^rd^ sensing step because the effective electron barrier height was reduced by the negative polarity of doxorubicin. Then, doxorubicin and Cu^2+^ ions were removed by deionized (DI) water to reuse the bio-FET with only the DNA receptor template (4^th^ erasing step), and the current level was returned to that of the bio-FET in the 1^st^ preparation step. This current level was similar in each cycle, and the overall changes in current level were also repeated for three detection cycles.

**The long-term stability of Cu^2+^-DNA/MoS_2_ based bio-FET in air**


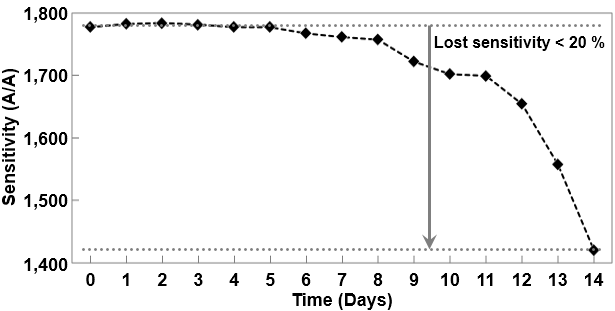


**Fig. S3.** The extracted sensitivity values of Cu^2+^-DNA/MoS_2_ based bio-FET as a function of air-exposure time.

To evaluate the long-term stability of the Cu^2+^-DNA/MoS_2_ based bio-FET, we monitored the sensitivity value of the device for two weeks. As shown in Figure S3, the extracted sensitivity value decreased over time, and we confirmed that the sensitivity was reduced about 20% after two weeks compared to the initial sensitivity. With this exposure time to air, moisture seems to degrade the detecting ability enhanced by Cu^2+^ ions.^S1^

[S1] Park, H.-Y. *et al.* n- and p-type doping phenomenon by artificial DNA and M-DNA on two-dimensional transition metal dichalcogenides. *ACS Nano* **8**, 11603-11613 (2014).

**The off-current level of Cu^2+^-DNA/MoS_2_ based bio-FETs in each step for ten detecting cycles**


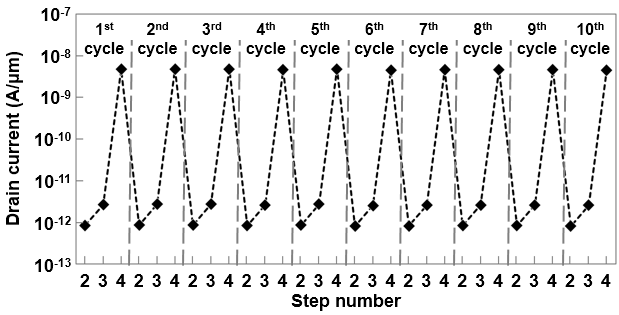


**Fig. S4.** The extracted off-current of Cu^2+^-DNA/MoS2 based bio-FETs in each step for ten detecting cycles.

We repeated the measurement of the Cu^2+^-DNA/MoS_2_ based bio-FET for ten cycles using the highest concentration of doxorubicin (50 μM). Then, we extracted the off-current values at V_GS_ = -30 V in each detecting cycle. As shown in Figure S4, the change in off-current level in each step was repeated for ten detecting cycles, showing its excellent reusability.

The comparison of biosensors in different types

| **Materials** | **Type of biosensor** | **Detection molecules**  **/range** | **Sensitivity** | **Reusability** | **Reference** |
| --- | --- | --- | --- | --- | --- |
| **MoS2** | Optical | Ag ion  /1 - 100 nM | Fluorescence intensity  700 - 1700 | No mention | Talanta 132, 658. (2015) |
|  | Optical | DNA  /0.5 - 130 nM | Fluorescence intensity  25 - 250 | No mention | Nanoscale 7, 2245. (2015) |
|  | Electrochemical  (FET) | pH  /3.0 - 9.0 pH | 713 A/A | No mention | ACS Nano 8, 3992. (2014) |
|  | Electrochemical  (CV and CA) | IgG-HRP  /0.1 - 20 ng/mL | Current density  0.2 - 1.4 μA/mm2 | No mention | RSC Adv. 5, 10134. (2015) |
| **WS2** | Electrochemical  (CV and CA) | 17β-estradiol  /10 pM - 5 nM | Current  35 - 40 μA | No mention | Anal. Methods 6, 8011. (2014) |
| **VS2** | Electrochemical  (CV and CA) | 17β-estradiol  /10 pM - 10 nM | Current  42 - 40 μA | No mention | Sens. Actuators B 201, 579. (2014) |
| **CoS** | Electrochemical  (CV and CA) | 17β-estradiol  /1 pM - 1 nM | Current  9.5 - 14 μA | No mention | Biosens. Bioelectron. 67, 184. (2015) |
| **CuS** | Electrochemical  (CV and CA) | DNA  /0.1 pM - 1 nM | Current  25 - 35 μA | No mention | Sens. Actuators B Chem. 209, 570. (2015) |
| **h-BN** | Electrochemical  (CV and CA) | Forchlorfenuron  /0.5 - 10 mM | Current  0.5 - 2.25 μA | Possible | Biosens. Bioelectron. 63, 294. (2015) |
| **Cyclodextrin-graphene** | Electrochemical  (CV and CA) | Doxorubicin  /10 - 200 nM | 1.4 | No mention | Electroanalysis 23, 2400. (2011) |
| **MoS2** | Electrochemical  (FET) | Doxorubicin  /0.1 nM - 50 μM | 1757.1 A/A | Possible | This work |

**Table S1.** Comparison of different types of biosensors.

The biosensors detected a wide-range of doxorubicin concentrations (0.1 nM − 50 μM for the Cu^2+^-DNA/MoS_2_ based bio-FET and 10 − 200 nM for the cyclodextrin-graphene based biosensor) and showed high sensitivity values (1757.1 for the Cu^2+^-DNA/MoS_2_-based bio-FET and 1.4 for the cyclodextrin-graphene-based biosensor). Furthermore, the Cu^2+^-DNA/MoS_2_-based bio-FET presented excellent reusability, which is a property that had not been investigated yet for most other biosensors.
